# Using Plant Functional Traits to Explain Diversity–Productivity Relationships

**DOI:** 10.1371/journal.pone.0036760

**Published:** 2012-05-18

**Authors:** Christiane Roscher, Jens Schumacher, Marlén Gubsch, Annett Lipowsky, Alexandra Weigelt, Nina Buchmann, Bernhard Schmid, Ernst-Detlef Schulze

**Affiliations:** 1 Department of Community Ecology, Helmholtz Centre for Environmental Research, UFZ, Halle, Germany; 2 Institute of Stochastics, Friedrich Schiller University Jena, Jena, Germany; 3 Institute of Agricultural Sciences, ETH Zurich, Zurich, Switzerland; 4 Max Planck Institute for Biogeochemistry, Jena, Germany; 5 Institute of Evolutionary Biology and Environmental Studies, University of Zurich, Zurich, Switzerland; 6 University of Leipzig, Institute of Biology, Leipzig, Germany; Lakehead University, Canada

## Abstract

**Background:**

The different hypotheses proposed to explain positive species richness–productivity relationships, i.e. selection effect and complementarity effect, imply that plant functional characteristics are at the core of a mechanistic understanding of biodiversity effects.

**Methodology/Principal Findings:**

We used two community-wide measures of plant functional composition, (1) community-weighted means of trait values (CWM) and (2) functional trait diversity based on Rao’s quadratic diversity (FD_Q_) to predict biomass production and measures of biodiversity effects in experimental grasslands (Jena Experiment) with different species richness (2, 4, 8, 16 and 60) and different functional group number and composition (1 to 4; legumes, grasses, small herbs, tall herbs) four years after establishment. Functional trait composition had a larger predictive power for community biomass and measures of biodiversitity effects (40–82% of explained variation) than species richness per se (<1–13% of explained variation). CWM explained a larger amount of variation in community biomass (80%) and net biodiversity effects (70%) than FD_Q_ (36 and 38% of explained variation respectively). FD_Q_ explained similar proportions of variation in complementarity effects (24%, positive relationship) and selection effects (28%, negative relationship) as CWM (27% of explained variation for both complementarity and selection effects), but for all response variables the combination of CWM and FD_Q_ led to significant model improvement compared to a separate consideration of different components of functional trait composition. Effects of FD_Q_ were mainly attributable to diversity in nutrient acquisition and life-history strategies. The large spectrum of traits contributing to positive effects of CWM on biomass production and net biodiversity effects indicated that effects of dominant species were associated with different trait combinations.

**Conclusions/Significance:**

Our results suggest that the identification of relevant traits and the relative impacts of functional identity of dominant species and functional diversity are essential for a mechanistic understanding of the role of plant diversity for ecosystem processes such as aboveground biomass production.

## Introduction

Rapid decline in biodiversity has motivated considerable research directed towards understanding how changes in biodiversity affect ecosystem functioning [1], [2]. Experimental biodiversity–ecosystem functioning research has demonstrated the importance of biodiversity for a number of ecosystem processes such as plant productivity, but it remains a central challenge to identify the underlying mechanisms [2]. Two mutually non-exclusive mechanisms are discussed as main drivers for greater plant productivity as a function of increasing plant diversity: The “sampling effect hypothesis” states that in experiments where species assemblages are randomly created, species-rich communities are more likely to include a species with disproportionate large effects on properties at the community-level [3], [4]. The “complementarity effect hypothesis” proposes that niche partitioning or facilitation among species allow for a more complete use of resources and therefore larger process rates at the community level [5], [6].

Mathematical partitioning of net biodiversity effects into complementarity and selection effects (the latter similar to sampling effects) as proposed by the “additive partitioning method” [7] has shown that mostly both effects contribute to positive species richness–productivity relationships in experimental grasslands (e.g. [7]–[11]). Selection as well as complementarity effects imply that ecosystem properties such as community productivity strongly depend on the functional characteristics of the constituent species. Functional traits are morphological, physiological and phenological features measurable at the individual level which modulate plant performance and individual fitness via their effects on growth, survival and reproductive output [12]. Plant traits largely determine how individual plant species contribute to processes at the community-level. However, plants need to balance a number of functional requirements; therefore the set of trait values realized by a species results from trade-offs integrating different functions and may reflect species-specific strategies.

Effects of functional traits on ecosystem properties have been quantified by two conceptually different approaches. On the one hand, community-weighted means of trait values ( = CWM) are usually calculated as mean trait values weighted by species relative abundances in a given community (e.g. [13], [14]). This measure quantifies the dominant trait values in a community and is consequently closely related to the “mass ratio hypothesis” [15] proposing that ecosystem processes are mainly determined by the functional traits of dominant species in a community (i.e. functional identity). CWM should therefore be related to sampling and selection effects although the definition of the second (in contrast to the first) originally does not require that species which mostly affect ecosystem properties achieve dominance [4].

On the other hand, a number of continuous measures have been developed which assess functional trait diversity of a community by quantifying the distribution of trait values among species (see reviews in [16], [17]). The concept of functional trait diversity is based on the assumption that with increasing trait dissimilarity among species the diversity in resource use strategies increases as well and species overlap along resource axes decreases [18]. Several criteria have been identified for a useful definition of functional diversity [16], [19], but all techniques sensitively depend on which traits are included in analyses [20]. Rao’s quadratic diversity FD_Q_ (Rao’s Q, [21]), which is the sum of pairwise functional distances between species weighted by their relative abundances, has been advocated repeatedly as a suitable measure for describing functional trait diversity (e.g. [22]–[24]). FD_Q_ should be closely related to complementarity effects and is largest when functionally different species, i.e. with large trait differences, reach similar high abundances [16].

Recent studies have shown that both, community-weighted means of trait values and functional trait diversity, can jointly explain variation in aboveground productivity in semi-natural grasslands [25]–[27]. In experimental grasslands, a higher community biomass has been shown to correlate positively with functional trait diversity [20], [28], [29] or a combination of functional trait diversity and community-weighted means of trait values [30]. However, so far none of the experimental studies considering species richness effects on productivity has performed a systematic analysis of how either community-weighted means of trait values or functional trait diversity may explain variation in productivity and the contribution of complementarity and selection effects on mixture performance. Here, we present a study carried out four years after establishment of a large grassland biodiversity experiment (Jena Experiment; [31]) comprising 66 mixtures of different species richness (2, 4, 8, 16, and 60 species) and different functional group number (1 to 4; grasses, legumes, small herbs, tall herbs) and respective monocultures of the 60 experimental species. Plant traits were measured in monocultures and additionally derived from the literature to characterize strategies of resource capture and use as well as life-history features of all species. The additive partitioning method [7] was used to partition net biodiversity effects into selection and complementarity effects. We applied a method proposed by Schumacher and Roscher [27] which is flexible in testing competing hypotheses on the effects of plant functional characteristics on ecosystem processes by using two different measures, i.e. community-weighted means of trait values (CWM) and functional trait diversity based on Rao’s quadratic diversity (FD_Q_). This method is based on the separate calculation of FD_Q_ in single traits which are used as candidate predictors in a multiple regression procedure, thus allowing for a weighting of different functional traits and maximizing their power to predict ecosystem processes. Firstly, we explored how the 60 experimental species assigned to different functional groups spread in a multivariate functional trait space to compare their resource acquisition and life-history strategies and trade-offs between different functions. Secondly, we tested how the experimental mixtures varied in their functional trait composition, i.e. CWM and FD_Q_, and how this varation in trait composition is related to sown species richness. Thirdly, we related community biomass production as well as measures of biodiversity effects and trait-based indices to assess whether community-weighted means of trait values (CWM), i.e. functional identity of dominant species, or functional trait diversity (FD_Q_), i.e. functional dissimilarity among species, are better predictors for high mixture performance.

## Results

### Species Characteristics in a Multivariate Trait Space

**Figure 1:**
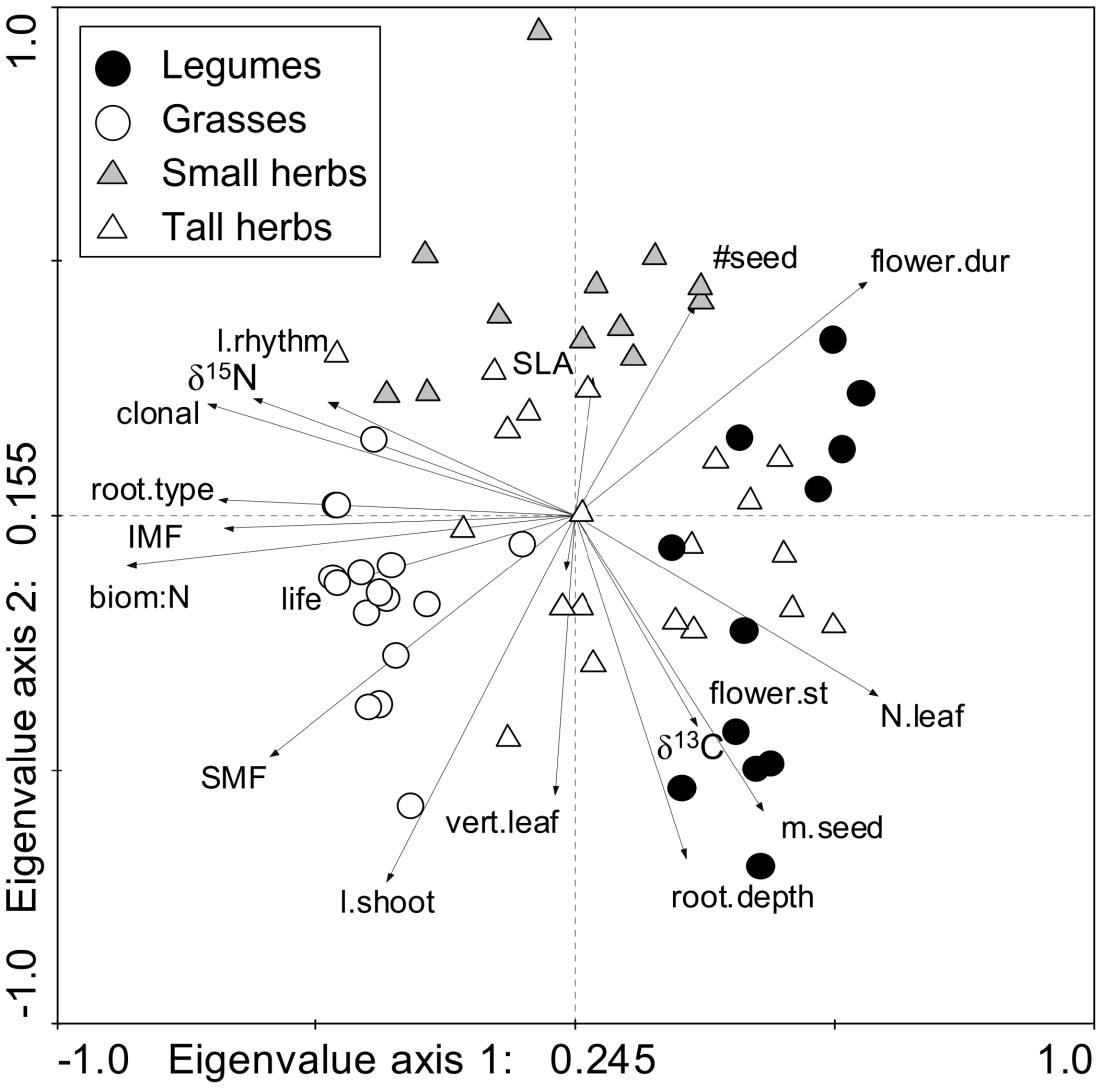
Standardized Principal Components Analysis (PCA; first vs. second axes) of 60 grassland species characterized by 18 functional traits. Plant traits were measured in species monocultures in the Jena Experiment and derived from the literature. Symbols show species assignment to plant functional groups. For list of variables and abbreviations see Table 2.

The two leading axes of a standardized PCA explained about 41% of variation (Fig. 1). Out of 153 correlations between traits 55 pairings were significant at P≤0.050. The first principal component accounting for 25% of species variation in multiple traits had high positive loadings for leaf nitrogen concentrations and length of the flowering period as well as high negative loadings for shoot biomass:N ratios, stem and inflorescence mass fractions, foliar δ^15^N values, rooting type and the species̀ ability for vegetative reproduction. In spite of considerable overlap among species assigned to different plant functional groups in the ordination space, the first principal component clearly separated legumes from grasses, while tall herbs and small herbs were more scattered between these functional groups (F_3,56_ = 38.50, P<0.001; Tukeỳs HSD test: P<0.001 for comparisons among legumes, grasses and herbs; P = 0.357 for comparison between small herbs and tall herbs). The second principal component accounted for 16% of species variation in multiple traits. This axis had high negative loadings for shoot length, seed mass, rooting depth and vertical leaf distribution. Small herbs which occupied the upper quadrants of the two-dimensional ordination space were separated from other pre-defined functional groups (F_3,56_ = 18.34, P<0.001; Tukeỳs HSD test: P<0.001 for comparisons of small herbs with grasses, legumes and tall herbs).

### Functional Trait Composition of Mixtures

**Figure 2:**
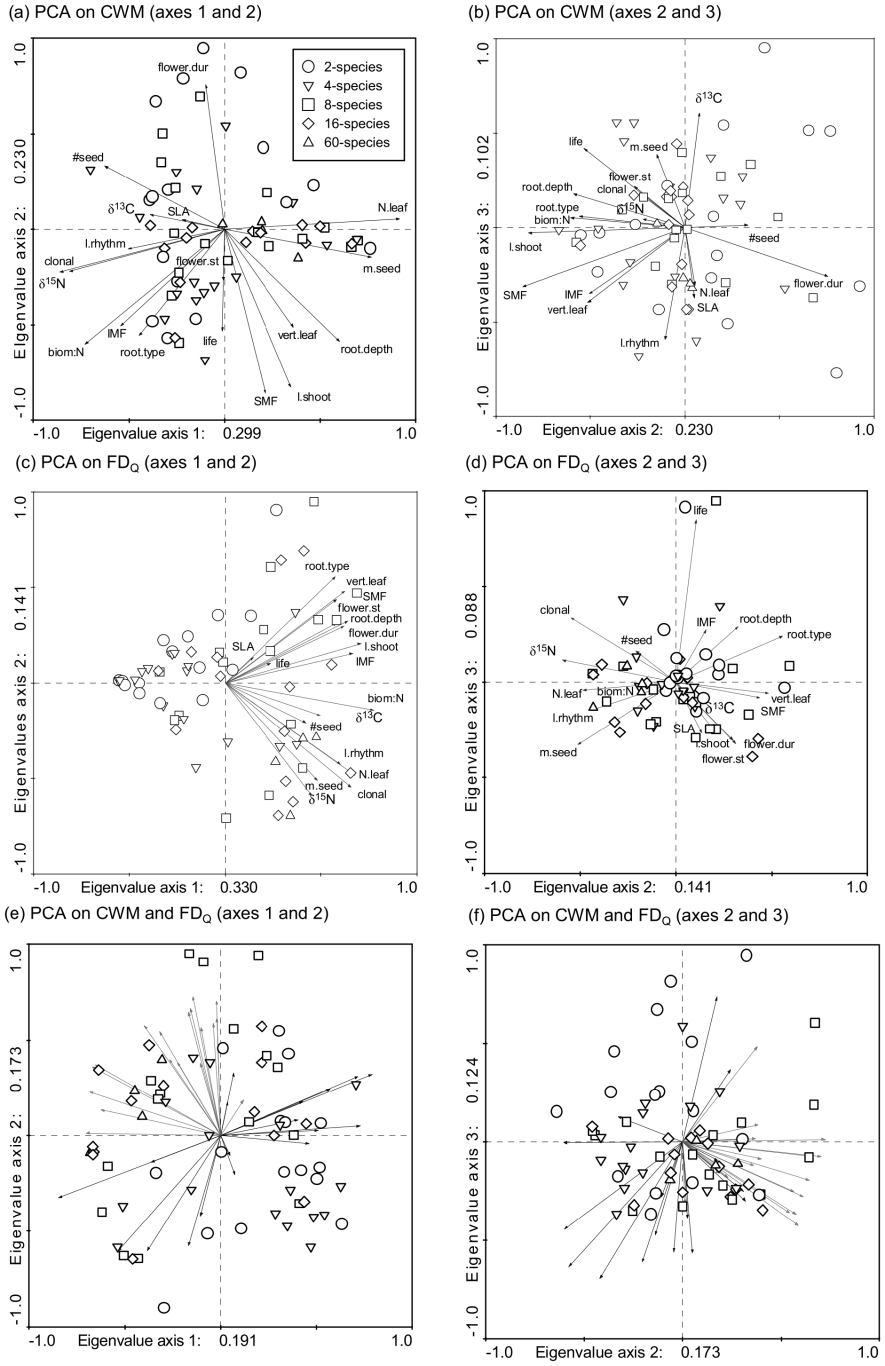
Standardized Principal Components Analysis (PCA) of community-weighted means of trait values (CWM) and functional trait diversity based on Raòs quadratic entropy (FD_Q_) of experimental grassland communities. PCA of community-weighted means of trait values (CWM) first vs. second axes (a), second vs. third axes (b), PCA of functional trait diversity (FD_Q_) first vs. second axes (c), second vs. third axes (d), and PCA of CWM and FD_Q_ in combination first vs. second axes (e), second vs. third axes (f). Community-weighted means of trait values (CWM) and functional trait diversity (FD_Q_) were calculated for each community based on species biomass proportions in the mixture and functional traits measured in species monocultures in the Jena Experiment and derived from the literature. Symbols show the position of each community sown with different numbers of species. Black arrows show CWM, red arrows show FD_Q_ for each trait.

The first principal component of a standardized PCA of community-weighted means of trait values (CWM) accounted for 30% of variation among mixtures. This axis had high positive loadings for CWM of leaf nitrogen concentrations, seed mass as well as rooting depth and high negative loading for CWM of foliar δ^15^N values, shoot biomass:N ratios, a species̀ ability for vegetative reproduction and seedling number (Fig. 2a). The second principal component explained 23% of variation in CWM among mixtures and was characterized by high positive loadings for CWM of length of the flowering period and high negative loadings for CWM of shoot length, stem mass fraction and shoot biomass:N ratios. The third principal component accounted for 10% of variation in CWM and had high positive loadings for foliar δ^13^C values (Fig. 2b). The first principal component of a standardized PCA of functional trait diversity (FD_Q_) explained 33% of variation in FD_Q_ among mixtures and had high positive loadings for a larger set of traits, e.g. FD_Q_ in shoot biomass:N ratios, shoot length, leaf nitrogen concentrations or species̀ ability for vegetative reproduction (Fig. 2c). The second principal component accounted for 14% of variation among mixtures and was characterized by high negative loadings for foliar δ^15^N values, while 9% of variation in FD_Q_ was explained by the third principal component that had high positive loadings for FD_Q_ in life cycle (Fig. 2d). Out of a total of 153 correlations in analyses of CWM and FD_Q_ respectively, approximately 50% of the pairings were significant at P≤0.050 (76 for CWM, 79 for FD_Q_). CWM and FD_Q_ were only weakly related (Fig. 2e, f), and only 55 pairings out of 324 correlations were significant. CWM of foliar δ^13^C values was significantly correlated with sown species richness, while FD_Q_ of 14 out of 18 traits had significant correlations with sown species richness.

### Aboveground Biomass Production

**Figure 3:**
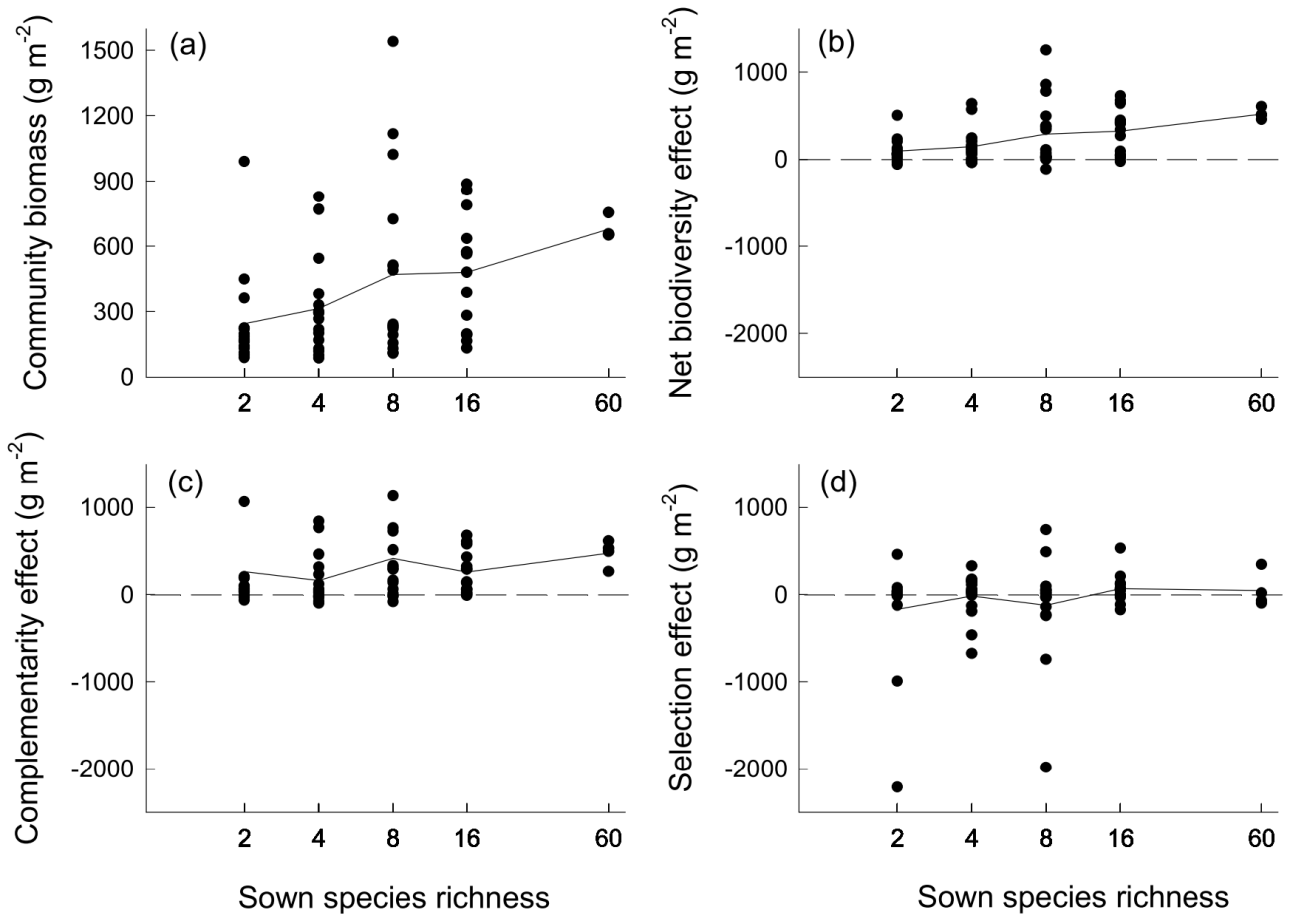
Aboveground community biomass, net biodiversity effect, complementarity effect, and selection effect as a function of species richness. Biomass data were recorded at estimated peak biomass in late May 2006 in the Jena Experiment. Lines show means across all mixtures per species-richness level.

Aboveground community biomass increased with increasing mixture species richness (R^2^ = 0.11; Fig. 3a). In separate models, CWM explained a larger proportion of variation in community biomass (R^2^ = 0.80; model A) than a model based on pure functional trait diversity effects (R^2^ = 0.36; model B, Table 1). In total, 7 out of 18 candidate traits were selected into the model best explaining variation in community biomass production purely based on CWM, indicating that communities containing dominant species with different trait combinations reached high productivity in our experiment. The chosen trait combinations included shoot length (long > short, i.e., longer shoot length was associated with higher community productivity), vertical leaf distribution (rosette > others), leaf nitrogen concentration (high > low), foliar δ^15^N values (low > high), ordering species along strategy spectra with regard to nitrogen and light acquisition and use; as well as life cycle (perennial > others), seasonality of foliage (summer- > wintergreen) and number of seedlings (low > high), ordering species along strategy spectra with regard to life-history and seasonal niche use. The proportion of explained variation only marginally increased in the model including CWM and FD_Q_ (R^2^ = 0.82; model C, Table 1) compared to the model purely based on CWM. Partial R^2^ values also provided evidence that CWM was the most important group of predictor variables in the combined model (Table 1). Although this result suggests that functional identity had the largest effects on community biomass production, FD_Q_ based on variability in foliar δ^15^N values had additional positive effects on community biomass production (model C, Table 1).

**Table 1:** Summary of best statistical models based on different groups of predictor variables (community-weighted means of trait values (CWM) and functional trait diversity (FD_Q_)) for community biomass, net biodiversity effects, complementarity effects and selection effects.

Response variable	R^2^	Selected traits	Partial R^2^	Selected traits		Partial R^2^
**Model A**		**Community weighted mean (CWM)**				
Community biomass	0.796	l.shoot, vert.leaf, N.leaf, δ^15^N, life, l.rhythm, #seed				
Net biodiversity effect	0.696	vert.leaf, N.leaf, δ^15^N, root.type, life, l.rhythm				
Complementarity effect	0.266	SLA, root type, #seed				
Selection effect	0.265	SLA, δ^15^N, root.type, m.seed				
**Model B**				**Functional trait diversity (FD_Q_)**		
Community biomass	0.356			l.rhythm, m.seed	positive	
Net biodiversity effect	0.378			N.leaf, δ^15^N, m.seed	positive	
Complementarity effect	0.236			N.leaf	positive	
Selection effect	0.284			SMF, N.leaf, life	negative	
**Model C**		**Community weighted means of trait values (CWM)**		**Functional trait diversity (FD_Q_)**		
Community biomass	0.818	l.shoot, vert.leaf, N.leaf, δ^15^N, life, l.rhythm, #seed	0.797	δ^15^N	positive	0.162
Net biodiversity effect	0.776	vert.leaf, N.leaf, δ^15^N, root.depth, root.type, life, l.rhythm	0.697	δ^15^N, clonal, IMF	positive	0.234
Complementarity effect	0.317	clonal	0.192	N.leaf	positive	0.236
Selection effect	0.396	δ^15^N, root.type, m.seed	0.252	N.leaf, life	negative	0.246

Analyses are based on 66 experimental mixtures of varying species richness (2, 4, 8, 16 and 60 species). For estimated coefficients see (Table S1, S2, S3), abbreviations of variable names are explained in Table 2.

**Table 2:** List of functional traits derived from measurements in monocultures of the Jena Experiment and from the literature.

	Variable	Abbreviation	Unit	Variable type	Description and trait categories	Source
**Resource acquisition and use**						
(1)	Shoot length	l.shoot	cm	continuous	stretched shoot length	measurement
(2)	Leaf distribution	vert.leaf		ordinal	vertical leaf distribution	literature
					(0) whole phytomass near theground (rosette)	
					(1) main part of phytomass nearground, but minor part along the stem	
					(2) equal parts of phytomass near the ground and along the stem	
(3)	Stem mass fraction	SMF	mg_stem_ mg^−1^_shoot_	continuous	dry mass of supporting tissue per total shoot mass	measurement
(4)	Specific leaf area	SLA	mm^2^_leaf_ mg^−1^_leaf_	continuous	leaf area per leaf dry mass	measurement
(5)	Foliar δ^13^C	δ^13^C	‰	continuous	^13^C isotopic signature of leaves	measurement
(6)	Leaf nitrogenconcentration	N.leaf	mg N g^−1^_leaf_	continuous	nitrogen mass per leaf dry mass	measurement
(7)	Biomass:N ratio	biom:N	g N g^−1^_shoot_	continuous	unit nitrogen per unit shoot mass	measurement
(8)	Foliar δ^15^N	δ^15^N	‰	continuous	^15^N isotopic signature of leaves	measurement
(9)	Depth of the rootsystem	root.depth		ordinal	(1) up to 20 cm	literature
					(2) up to 40 cm	
					(3) up to 60 cm	
					(4) up to 100 cm	
					(5) >100 cm	
(10)	Type of the root system	root.type		ordinal	(1) long-living primary root system (beet- or stake-like taproots)	literature
					(2) secondary fibrous roots in addition to the primary root system	
					(3) short-living primary root system, extensive secondary root system	
**Life history characteristics**						
(11)	Life cycle	life		ordinal	(1) annual	literature
					(2) biennial or monocarpic perennial	
					(3) perennial	
(12)	Clonal growth	clonal		ordinal	(0) no clonal growth	literature
					(1) clonal growth	
(13)	Seasonality of foliage	l.rhythm		ordinal	(1) deciduous	literature
					(2) partly deciduous (most foliage dies off in winter)	
					(3) evergreen (all-season with foliage)	
(14)	Start of flowering period	flower.st		ordinal	(1) before May	literature
					(2) May	
					(3) June	
					(4) July	
(15)	Duration of flowering period	flower.dur		ordinal	(1) two months or less	literature
					(2) three months	
					(3) four months	
					(4) more than 4 months	
(16)	Inflorescence massfraction	IMF	mg_inflorescence_ mg^−1^_shoot_	continuous	dry mass of reproductive organs per total shoot mass	measurement
(17)	Seed mass	m.seed	mg	continuous	average seed mass	measurement
(18)	Seedling number	#seed	m^−2^	continuous	number of emerged seedlings based on three census	measurement

### Measures of Biodiversity Effects

The net biodiversity effect (NE) was positive across all species-richness levels (test for overall mean≠0: F_1,61_ = 56.03, P<0.001). Increasing mixture species richness had positive effects on NE (R^2^ = 0.13). In separate models, CWM explained a larger proportion of variation in net biodiversity effects (NE) (R^2^ = 0.70) than did FD_Q_ (R^2^ = 0.38). The proportion of explained variation increased when models based on pure effects of CWM were extended with FD_Q_ (R^2^ = 0.78; model C). A combination of traits characterising efforts in nutrient acquisition (high leaf nitrogen concentrations, low foliar δ^15^N values, large rooting depth and extensive secondary root system), rosette-like vertical leaf distribution, summer-green foliage and perennial life cycle were selected for CWM, while variability in foliar δ^15^N values, in species’ ability for clonal reproduction and in investment into reproductive structures were selected for FD_Q_. The comparison of partial R^2^ values showed that effects of the traits selected for CWM exceeded effects of traits selected for FD_Q_ in explaining variation in net biodiversity effects (Table 1).

The complementarity effect (CE) was positive across all species-richness levels (test for overall mean≠0: F_1,61_ = 28.78, P<0.001; Fig. 3c), while the selection effect did not differ significantly from zero (test for overall mean≠0: F_1,61_ = 1.13, P = 0.293; Fig. 3d). An increasing species richness of mixtures did not affect CE and SE. Positive effects of FD_Q_ based on variabililty in leaf nitrogen concentrations in the pure trait-diversity model explained a similar proportion of variance (R^2^ = 0.24; model B) in the complementarity effect (CE) as the model solely based on CWM (R^2^ = 0.27; model A), which included community-weighted means in SLA, root type and seedling number. The proportion of explained variation in CE increased in the model combining CWM and FD_Q_ (R^2^ = 0.32; model C, Table 1). This model included negative effects for the ability for clonal reproduction as CWM, and positive effects of diversity in leaf nitrogen concentrations. Partial R^2^ values indicated that FD_Q_ was more important than CWM in explaining variation in CE, when both groups of predictor variables were analysed in combination (Table 1).

CWM (R^2^ = 0.27; model A) and FD_Q_ (R^2^ = 0.28; model B) also explained a similar proportion of variation in the selection effect (SE) when used separately as predictors (Table 1). The best model explaining variation in SE contained both groups of predictor variables (R^2^ = 0.40; model C). Low foliar δ^15^N values, extensive secondary root system and large seed mass as CWMs were correlated with positive selection effects, while variability in leaf nitrogen concentrations and life cycle as FD_Q_ were correlated with negative selection effects (Table 1).

## Discussion

The increasing consensus that ecosystem processes are governed by the functional traits of species, their abundance and distribution in a community [32], [33] has attracted growing attention on the use of functional trait composition, rather than species richness, in the exploration of biodiversity–ecosystem functioning relationships. Community-weighted means of trait values (CWM) characterising the functional identity of dominant species and different metrics of functional trait diversity describing the average functional dissimilarity among species, have rarely been incorporated in combined analyses in natural ecosystems (but see [25]–[27]) and have only recently been tested in a biodiversity experiment [30]. Our analyses of CWM and FD_Q_ based on a large spectrum of functional traits in a grassland biodiversity experiment (Jena Experiment) provided evidence that both reflect different components of functional trait composition (Fig. 2e, f). The step-wise modelling procedure including both groups of predictor variables (CWM, FD_Q_) applied to explore their relative contribution in explaining variation in biomass production and measures of biodiversity effects showed that in all cases the combination of CWM and FD_Q_ resulted in models with the largest explanatory power, but the relative importance of both components of functional trait composition depended on the analysed response variable: CWM explained a larger proportion of variation in community biomass and net biodiversity effects than did FD_Q_, whereas FD_Q_ explained a similar amount of variation in complementarity and (negative) selection effects as did CWM for variation in complementarity and (positive) selection effects. Thus, our results clearly demonstrate the need to unite the conceptually separate consideration of functional identity and functional diversity to further advance the understanding of mechanisms underlying biodiversity–ecosystem functioning relationships.

### Species Characteristics in Multiple Traits

Although the 60 grassland species substantially differed in their functional trait combinations (Fig. 1), the first principal component separated species according to characteristics of their “nutrient economy”, i.e. the capture and use of nitrogen. This axis of variation represented a gradient of species differing in their leaf nitrogen concentrations and δ^15^N values, shoot biomass:N ratios, root type, seasonality of foliage and ability for clonal reproduction. It differentiated species based on a functional trade-off in a set of plant attributes that are either directed to optimize rapid resource acquisition or permitting the conservation of resources, which is largely congruent to previous work at different geographic scales or locations (e.g. [34]–[36]). This trait axis distinguished the *a priori* defined functional groups of grasses from legumes, while non-leguminous herbaceous species had intermediate positions. The second principal component was related to aboveground plant size and structure (e.g. shoot length, stem mass fraction, vertical leaf distribution) and rooting depth. A larger plant size correlated positively with seed mass, which is consistent with results from large-scale studies [37]. A trade-off in strategies of generative reproduction distinguished species with heavier seeds and a later starting, short period of flowering compared to species with a longer, earlier starting flowering period and a larger number of emerging seedlings. A larger seed mass generally conveys benefit in seedling establishment [38]. Therefore, the higher number of seedlings in small-seeded species is likely to be based on a trade-off between seed mass and seed number, i.e. an inverse relation between seed production and seed size [39].

### The Role of Functional Traits in Explaining Variation in Community Biomass Production and Measures of Biodiversity Effects

In natural communities, species are not assembled randomly from a local species pool; thus particular ecological traits should be selected for in the process of community assembly [40]. Biodiversity experiments have been criticized for their random species selection, ignoring the impact of abiotic and biotic filters which constrain diversity in natural ecosystems [41]–[43]. Jiang et al. [43] argue that in natural communities abundant species are more likely to be widespread, thus increasing the chance for a positive selection effect. The design of the Jena Experiment ensures that the number of functional groups is near-orthogonally crossed with species richness [31]. Species combinations were randomly chosen from the respective functional groups, which increases the chance for each member of the species pool to be more frequent in multi-species communities irrespective of its potential to become dominant. Nevertheless, our analyses showed that increasing community biomass production was best explained by CWM, indicating that traits of the dominant species were most important for high productivity. The selection of CWM in high foliar nitrogen concentrations and low foliar δ^15^N values, which characterise legumes (Fig. 1; Table S3) showed that N_2_-fixing legumes had positive effects on community biomass production. However, the additional positive effects of diversity in foliar δ^15^N values suggest that a higher diversity in nitrogen acquisition strategies increased community biomass. Our analyses also showed that not only abundance-weighted means of leaf nitrogen concentrations and foliar δ^15^N values, but also CWM of a larger suite of traits (Table 1), which were widely spread in the multiple trait space (Fig. 1), explained variation in community biomass. Additional analyses (not shown) to evaluate whether high CWMs in different traits were associated with the occurrence of different species, provided evidence that three legume species, i.e. *Lathyrus pratensis* L., *Medicago lupulina* L., *Onobrychis viciifolia* Scop., were not only associated with CWM in nitrogen-related traits, but also with CWM characterising life cycle (low seedling number, deciduous leaves, and no ability for vegetative reproduction in case of *M. lupulina*, *O. viciifolia*) and shoot length (*O. viciifolia*). In contrast, other species were associated with CWM in single traits, e.g. low values for CWM in vertical leaf distribution with the occurrence of the rosette plants *Plantago media* L. and *Primula veris* L., large CWM in shoot length with the occurrence of the grass species *Bromus erectus* Huds. and *Dactylis glomerata* L.

In semi-natural grasslands, a tall growth usually associated with competitive dominance [44], has been shown to be related with high aboveground biomass production [27], [42]. High community mean values of shoot length also contributed to high community biomass in our experimental grasslands, but in addition leaf distribution along the stem (in favour of rosette plants) and life-history traits such as a perennial life cycle, seasonality of foliage (favouring summergreen species) and a low reproduction by seeds characterized communities with high biomass production. Due to the random allocation, species with traits favouring dominance in semi-natural grasslands did not necessarily have a larger probability to be present in each experimental multi-species mixture. Contrarily, species with traits which would not correlate with a potential for dominance in natural environments may achieve higher abundances in these artificially created experimental grasslands because of a reduced likelihood for competitive exclusion [43].

A higher importance of CWM and additional positive effects of FD_Q_ with a similar spectrum of selected traits also explained variation in net biodiversity effects best. The replacement of shoot length by root characteristics (i.e. larger rooting depth and a higher proportion of species with an extensive secondary root system which usually possess a higher root density [45]) suggested that the exploitation of soil resources is important for positive net biodiversity effects. The selection effect (Fig. 3d) was best explained by a combination of negative functional trait diversity effects and CWM. Higher selection effects were positively correlated with high mean values of traits associated either with particular resource acquisition strategies (grasses with an extensive secondary root system, N_2_-fixing legumes with low δ^15^N values and high seed mass, Fig. 1), when combined with a low diversity of traits associated with nitrogen use (leaf nitrogen concentrations), light acquisition (stem mass fraction) and life cycle. In contrast, a combination of CWM and positive trait diversity effects explained variation in complementarity effects best. Positive effects of legume presence on these measures have been reported from several biodiversity experiments including the Jena Experiment (e.g. [46]–[48]). The incorporation of diversity in leaf nitrogen concentrations and CWM in species̀ ability for vegetative spread (low ability = characteristic of several herbs, Fig. 1) emphasized that not only the presence of N_2_ fixing legumes with high foliar nitrogen concentrations (Fig. 1), but species separation along trait axes associated with nitrogen acquisition is important for positive complementarity effects among grassland species.

In summary, our results show that traits may vary considerably with respect to their relative importance as weighted mean trait values (CWM) or trait variance (FD_Q_). Traits associated with nitrogen acquisition and use as foliar δ^15^N values and leaf nitrogen concentrations were incorporated as FD_Q_ and CWM in our models, indicating that both diversity in strategies of nitrogen acquisition and use (related to high FD_Q_) and high abundances of legumes (expressed by high CWM in leaf nitrogen concentrations and low CWM in foliar δ^15^N values) are essential for a high community biomass, positive complementarity and net diversity effects in our study system. The larger set of morphological and life-history traits which were additionally incorporated as CWM, however, suggested that species identity expressed as high proportions of species with different trait combinations are important for a high performance of particular mixtures randomly assembled from an experimental species pool.

### Methodological Aspects

The importance of considering the functional diversity with respect to individual traits when trying to explore biodiversity–ecosystem functioning relationships was recently emphasized by Spasojevic and Suding [49]. The calculation of functional diversity indices is usually based on multiple traits. This approach is justified particularly at larger environmental scales, where plant strategies are reflected in functional trade-offs and a coordinated variation of functional traits [34], [50]. However, our analysis of 60 grassland species (Fig. 1) provided evidence for a considerable variation in their functional trait combinations. Therefore, the incorporation of multiple traits in single multivariate indices of functional diversity increases the chance to include functional traits that are either irrelevant or have opposing effects which may mask important diversity effects. In addition, single multivariate functional diversity indices do not directly permit the identification of functional traits relevant for a particular ecosystem process. So far this problem has been circumvented by comparing alternative models with different trait combinations [20], [26]. We extended this approach by estimating relative weights for individual traits [27]. The consideration of variability of each functional trait cannot fully avoid multicollinearity among predictors and does not allow for an unequivocal partitioning of sources of variability in ecosystem processes. However, our approach of deriving the importance of diversity with respect to different functional traits by estimating relative weights implements a practical procedure to identify the relevant traits. Despite the potential multicollinearity problems, the statistical process of trait selection (CWM and FD_Q_) for our best models seems to be relatively stable. Comparing sets of three best models (see Tables S1, S2, S3), the combinations of selected traits were always quite similar and replacements occurred with traits which were closely related as revealed by detailed PCA (Fig. 2). Although FD_Q_ is not necessarily expected to strongly correlate with species richness and may achieve maximum values with two species only [16], the diversity of most traits was positively correlated with species richness in our experiment. Therefore, we expect that our approach would be even more useful in identifying the relevant components of diversity in natural ecosystems.

### Conclusions

A number of recent studies on biodiversity–ecosystem functioning relationships have suggested that the functional composition is a main driver of ecosystem processes, but so far the relative effects of functional identity (or community-weighted mean traits) and trait variation (or functional diversity) remained mostly untested (but see [25]–[27], [30]) and the identification of relevant functional traits for different ecosystem processes has largely been ignored. Our results clearly emphasize the need to incorporate different aspects of functional composition (functional identity, functional diversity) in studies of biodiversity–ecosystem functioning relationships. In addition, the large dimensionality in species̀ functional trait composition and the differential importance of single traits imply that the identification of key functional traits may contribute to a better understanding of the drivers of different ecosystem processes. Thus, our modelling framework may contribute to resolve the controversy about mechanisms behind biodiversity–ecosystem functioning relationships in experimental biodiversity research, but it is also a tool to identify the relevant components of biodiversity for the maintenance of ecosystem functioning in natural systems.

## Materials and Methods

### Study Area and Experimental Design

The Jena Experiment was established in spring 2002 on former arable land located in the floodplain of the river Saale near the city of Jena (Thuringia, Germany; 50°55′ N, 11°35′ E, 130 m a.s.l.). The soil of the experimental site is a Eutric Fluvisol developed from up to 2 m-thick loamy fluvial sediments. Soil texture ranges from sandy loam near the river to silty clay with increasing distance from the river. The area around Jena has a mean annual air temperature of 9.3°C, mean annual precipitation is 587 mm [51].

The experimental species pool comprised 60 plant species common to Central European mesophilic grasslands [52], which were divided into four functional groups: 16 grasses, 12 small herbs, 20 tall herbs and 12 legumes. In total, 82 plots of 20×20 m size were established. These plots vary in species richness (1, 2, 4, 8, 16 and 60) and functional group number (1 to 4) and composition, whereby for each functional group composition as many species richness levels as possible had been designed. In total, each species-richness level had 16 plots, except for the 16-species level with 14 plots because not enough species were available to create pure legume and small herb mixtures at this species-richness level. The 60-species mixture was established on 4 plots. Species were randomly selected from the species pool for each particular community. In addition, each experimental species was sown in two replicated smaller monocultures of 3.5×3.5 m size. Initial sowing density amounted to 1000 viable seeds per m^2^. In mixtures, all species were sown with equal proportions (for further details, see [31]). To account for the gradient in soil texture, the experimental area was divided into four blocks parallel to the river, each containing an equal number of large plots per species-richness level and of small monocultures per functional group. All plots were mown twice per year in early June and September as it is typical for the management of extensive hay meadows in the region. No fertilizer was applied during the experiment. Biannual weeding campaigns (April, July) served to maintain the sown species combinations. Management was organized block-wise.

### Data Collection

#### Aboveground biomass production

Aboveground biomass production was assessed by cutting plants 3 cm above ground level at estimated peak biomass in late May 2006. Four samples were harvested in large mixture plots, and two samples were taken in small monocultures in randomly selected subplots (20×50 cm size), excluding the outer 70 cm of the plots. Clipped plant material was sorted to species sown into a particular community, removing unsown species and detached dead material. Biomass of all samples was determined after drying (48 h, 70°C). For further details, see [53]. Aboveground community biomass was calculated for each experimental community as the mean of replicated samples per plot.

#### Trait data

Trait data were collected in monocultures of each species in late May 2006 (corresponding to the time of biomass harvest) with the exception of six species, which were sampled in May 2008 or 2009 (*Anthriscus sylvestris* (L.) Hoffm., *Bromus hordeaceus* L., *Cynosurus cristatus* L., *Holcus lanatus* L., *Pastinaca sativa* L., *Sanguisorba officinalis* L.). Three species could not be studied in monoculture because they went extinct (*Cardamine pratensis* L.) or their abundance was too low for destructive sampling (*Campanula patula* L., *Luzula campestris* (L.) Dc.). Thus, trait data for these species were recorded in a low-diversity mixture, where these species were present. Single shoots were the basic unit for all measurements because the vegetative spread of several species with above- or belowground runners hampered the clear identification of plant individuals in some cases. Shoots rooting closest to regularly spaced points along a transect (each 25 cm excluding the outer 70 cm of the plot margin) were chosen and cut off at ground level. Shoots were immediately put into sealed plastic bags and stored in a cool box. In the laboratory, maximum stretched shoot length was measured. Then, shoots were separated into biomass components: stems (including leaf sheaths in case of grasses and secondary axes in case of herbs), leaves (being leaf blades in case of grasses, including petioles and rhachis in case of herbs with compound leaves) and reproductive parts (flowers, fruits). A leaf area meter (LI-3100 Area Meter, Li-COR, Lincoln, USA) was used to determine the area of three to four fully developed leaves (leaf blades in case of grasses) per shoot. All harvested material was dried at 70°C (48 h) and weighed. For subsequent chemical analyses, the dry material of measured leaves and bulk samples of the residual shoot material of each species were pooled per plot and ground to a fine powder. Nitrogen and carbon concentrations of the bulk samples were measured with an elemental analyzer (Vario EL Element Analyzer, Elementar, Hanau, Germany). Nitrogen concentrations as well as nitrogen and carbon isotope ratios (δ^15^N and δ^13^C, respectively) were determined from leaf samples with an isotope-ratio mass spectrometer (IRMS, Delta ^plus^ XP and Delta C prototype Finnigan MAT respectively, Bremen, Germany).

Seedlings (plant individuals with cotyledons) were counted three times in 2006 (April, July, October) in all small monoculture plots to account for species-specific differences of seedling emergence. For each census, three quadrats (30×30 cm) were randomly placed and all emerged seedlings of the respective species were counted. Cumulative seedling densities per m^2^ were calculated for each monoculture based on pooled data from all census dates.

Data from species replicates were averaged when trait data in both monoculture plots per species were sampled. Average seed mass was determined by weighing 5 batches of 50 seeds per species from seed material purchased from commercial suppliers (Rieger-Hofmann GmbH, Blaufelden-Raboldshausen, Germany) used for the establishment of the Jena Experiment. Further species attributes characterising belowground morphology and life history were derived from the literature [54]–[56] as categorical variables, resulting in a matrix of 18 plant functional traits in total (see Table 2).

### Data Analyses

#### Aboveground biomass production and measures of biodiversity effects

Aboveground community biomass at the time of harvest was calculated for each experimental community as the mean of the four samples per large plot and the two samples per small monoculture plot. Net biodiversity effects (NE), complementarity effects (CE) and selection effects (SE) were calculated for each mixture using the additive partitioning method [7]. The net biodiversity is defined as
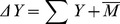
(1)where Σ*Y* is the summed observed biomass for each species in a mixture (i.e. community biomass) and 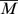 is the average monoculture biomass of all species in this mixture. The net biodiversity effect can be partitioned into two additive components.



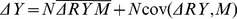
(2)The selection effect is quantified as 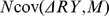, where *N* is the number of species in mixture, *M* is a species̀ monoculture biomass, and *ΔRY* is the difference between the observed relative yield (*Y/M*) and the expected relative yield (i.e. its sown proportion *1/N*). The complementarity effect is measured as 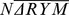, where 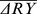 is the average *ΔRY* of all species in the mixture. Biomass data of the two small monocultures per species were averaged for all calculations.

#### Community-weighted means of trait values and functional trait diversity

Community-weighted means of trait values (CWM) were calculated for each community based on species biomass proportions according to the equation
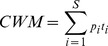
(3)where *S* is the number of species in the community, *p_i_* are the species biomass proportions and *t_i_* are species-specific trait values.

Functional trait diversity was computed as Raòs quadratic entropy (FD_Q_)
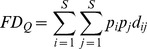
(4)where *S* is the number of species in the community, *p_i_* and *p_j_* are the relative abundances of species *i* and *j,* and *d_ij_* is the trait distance between species *i* and *j* in the community. Data on leaf nitrogen concentration, seed mass, seedling number, inflorescence mass fraction, foliar δ^15^N and stretched shoot length were log-transformed prior to analysis to meet the assumptions of normality.

#### Species and community characteristics in multivariate trait space

A standardized principal components analysis (PCA) was performed to explore species differences in multiple trait space. In addition, standardized PCAs were applied to investigate multiple relationships between mixture CWM and FD_Q_ based on single traits and species biomass proportions in mixture.

#### Predicting aboveground biomass production and measures of biodiversity effects from plant functional traits

FD_Q_ can be interpreted as the average dissimilarity of two randomly chosen individuals from the community, thus comprising information about functional richness and functional evenness of a community [57]. Since the trait distances *d_ij_* strongly depend on which functional traits are incorporated and on the chosen distances measure, FD_Q_ comprises a large number of possible functional diversity measures. This flexibility has been used to identify those functional traits whose diversity is most relevant for particular ecosystem processes by comparing the predictive performance of FD_Q_ based on different suites of traits [20], [26]. When based on multiple traits and the squared Euclidean distance as a dissimilarity measure, FD_Q_ represents the sum of variances of individual plant traits, thus relating FD_Q_ to a common measure of variability of single traits [19], [27]. Exploiting this additivity with respect to traits, the qualitative exclusion/inclusion of traits has recently been generalized to a more quantitative approach where additionally relative weights for the different traits can be estimated [27]. Using this approach FD_Q_ can be interpreted as a weighted average of functional diversity measures based on single traits (see Text S1, Supporting Information for technical details). To allow the interpretation of weights as measures of relative importance of traits all traits were standardized to unit variance prior to calculating FD_Q_.

To study the relative contribution of community-weighted means of trait values and functional diversity to the amount of explained variation in aboveground biomass production and measures of biodiversity effects we considered statistical models with different combinations of explanatory variables (A) CWM, (B) FD_Q_, and (C) CWM and FD_Q_. Within each class of models we selected the best fit based on leave-one-out cross validation. Detailed information on the three best models can be found in the (Table S1, S2, S3). The coefficient of determination R^2^ is given as a summary measure for explained variation.

All data analyses were performed with the statistical software R 2.11.1 (R Development Core Team, http://www.R-project.org) and the implemented package *leaps* [58] and *quadprog* [59], and CANOCO 4.5 [60] was used for PCA.

## Supporting Information

Table S1 Summary of the best three models based on CWM.
(DOC)

Table S2 Summary of the best three models based on FD_Q._
(DOC)

Table S3 Summary of the best three models based on CWM and FD_Q._
(DOC)

Text S1 Detailed method description on estimating the relative importance of functional traits.
(PDF)
