# Supplementary material for: Using Plant Functional Traits to Explain Diversity–Productivity Relationships
Source: PLoS One. 2012 May 18;7(5):e36760. doi: 10.1371/journal.pone.0036760 (PMC3356333; doi:10.1371/journal.pone.0036760)
Supplement: Table S1 — Summary of the best three models based on CWM. (DOC) [file pone.0036760.s001.doc]

**TableS1** Summary of the best three models based on CWM

| Response | Model 1 |  | Model 2 |  | Model 3 |  |
| --- | --- | --- | --- | --- | --- | --- |
|  | Selected traits | Estimated Parameters | Selected traits | Estimated Parameters | Selected traits | Estimated Parameters |
| Biomass | Intercept | -1372.45 | Intercept | -1051.79 | Intercept | -1251.89 |
|  | l.shoot | 122.45 | l.shoot | 94.62 | l.shoot | 120.32 |
|  | vert.leaf | 148.75 | SLA | -10.82 | vert.leaf | -126.76 |
|  | N.leaf | 470.21 | N.leaf | 428.17 | N.leaf | 443.20 |
|  | δ^15^N | -256.40 | δ^15^N | -293.21 | δ^15^N | -278.23 |
|  | life | 201.47 | life | 238.10 | life | 209.83 |
|  | l.rhythm | -106.78 | l.rhythm | -114.85 | l.rhythm | -104.82 |
|  | #seed | -21.43 | flower.st | 65.63 | flower.st | 22.26 |
|  |  |  |  |  | #seed | -19.73 |
|  |  | R^2^=0.796 |  | R^2^=0.795 |  | R^2^=0.798 |
| NE | Intercept | -1788.69 | Intercept | -1728.29 | Intercept | -1591.78 |
|  | vert.leaf | -132.59 | vert.leaf | 152.38 | vert.leaf | -136.23 |
|  | N.leaf | 575.58 | SMF | 130.73 | N.leaf | 573.83 |
|  | δ^15^N | -201.21 | N.leaf | 568.10 | δ^15^N | -154.96 |
|  | root.type | 86.07 | δ^15^N | -183.25 | life | 219.35 |
|  | life | 216.83 | root.type | 66.65 | l.rhythm | -109.40 |
|  | l.rhythm | -119.43 | life | 202.78 | flower.dur | -31.61 |
|  |  |  | l.rhythm | -125.31 |  |  |
|  |  |  |  |  |  |  |
|  |  | R^2^=0.696 |  | R^2^=0.701 |  | R^2^=0.694 |
| CE | Intercept | 1191.06 | Intercept | 1229.38 | Intercept | 1296.92 |
|  | SLA | 30.63 | SLA | 24.75 | SLA | 31.43 |
|  | root.type | -517.00 | root.type | -389.65 | δ^15^N | 329.20 |
|  | #seed | -75.20 | clonal | -326.87 | root.type | -711.29 |
|  |  |  | m.seed | -92.30 | #seed | -104.60 |
|  |  |  | #seed | -62.90 |  |  |
|  |  | R^2^=0.266 |  | R^2^ = 0.311 |  | R^2^=0.295 |
| SE | Intercept | -457.63 | Intercept | -805.29 | Intercept | -301.07 |
|  | SLA | -19.36 | δ^15^N | -365.50 | SLA | -19.48 |
|  | δ^15^N | -367.04 | root.type | 498.65 | δ^15^N | -523.51 |
|  | root.type | 500.82 | m.seed | 114.10 | root.type | 404.40 |
|  | m.seed | 102.87 |  |  | clonal | 382.03 |
|  |  |  |  |  | m.seed | 136.02 |
|  |  | R^2^=0.265 |  | R^2^=0.252 |  | R^2^=0.308 |

For abbreviations of variable names see Table 2.
