# Supplementary material for: Using Plant Functional Traits to Explain Diversity–Productivity Relationships
Source: PLoS One. 2012 May 18;7(5):e36760. doi: 10.1371/journal.pone.0036760 (PMC3356333; doi:10.1371/journal.pone.0036760)
Supplement: Table S2 — Summary of the best three models based on FDQ. (DOC) [file pone.0036760.s002.doc]

| Response | Model 1 |  |  | Model 2 |  |  | Model 3 |  |  |
| --- | --- | --- | --- | --- | --- | --- | --- | --- | --- |
|  | Selected traits | Estimates | rel. weight | Selected traits | Estimates | rel. weight | Selected traits | Estimates | rel. weight |
| Biomass | Intercept | 190.32 |  | Intercept | 182.79 |  | Intercept | 221.85 |  |
|  | l.rhythm | 81.16 | 0.83 | δ^15^N | 26.23 | 0.50 | m.seed | 149.72 | 1.00 |
|  | m.seed | 118.47 | 1.00 | l.rhythm | 72.87 | 0.82 |  |  |  |
|  |  |  |  | m.seed | 106.72 | 1.00 |  |  |  |
|  |  | R^2^=0.356 |  |  | R^2^=0.364 |  |  | R^2^=0.331 |  |
| NE | Intercept | 46.07 |  | Intercept | 46.50 |  | Intercept | 57.32 |  |
|  | N.leaf | 29.22 | 0.54 | δ^15^N | 30.84 | 0.54 | δ^15^N | 43.27 | 0.62 |
|  | δ^15^N | 36.66 | 0.60 | clonal | 33.85 | 0.57 | m.seed | 110.96 | 1.00 |
|  | m.seed | 101.33 | 1.00 | m.seed | 105.70 | 1.00 |  |  |  |
|  |  | R^2^=0.378 |  |  | R^2^=0.377 |  |  | R^2^=0.367 |  |
| CE | Intercept | 102.12 |  | Intercept | 53.31 |  | Intercept | 41.19 |  |
|  | N.leaf | 184.23 | 1.00 | SMF | 65.19 |  | N.leaf | 168.84 |  |
|  |  |  |  | N.leaf | 179.53 |  | #seed | 75.31 |  |
|  |  | R^2^=0.236 |  |  | R^2^=0.253 |  |  | R^2^=0.266 |  |
| SE | Intercept | 175.05 |  | Intercept | 163.51 |  | Intercept | 191.59 |  |
|  | SMF | -101.84 | 0.83 | SMF | -101.11 | 0.81 | SMF | -98.40 | 0.81 |
|  | N.leaf | -71.93 | 0.70 | δ^13^C | -114.36 | 0.86 | SLA | -26.51 | 0.42 |
|  | life | -147.34 | 1.00 | life | -154.34 | 1.00 | N.leaf | -70.75 | 0.69 |
|  |  |  |  |  |  |  | life | -148.16 | 1.00 |
|  |  | R^2^=0.284 |  |  | R^2^=0.277 |  |  | R^2^=0.287 |  |

**Table S2** Summary of best three models based on FD_Q_

For abbreviations of variable names see Table 2.
