# Supplementary material for: Using Plant Functional Traits to Explain Diversity–Productivity Relationships
Source: PLoS One. 2012 May 18;7(5):e36760. doi: 10.1371/journal.pone.0036760 (PMC3356333; doi:10.1371/journal.pone.0036760)
Supplement: Table S3 — Summary of the best three models based on CWM and FDQ. (DOC) [file pone.0036760.s003.doc]

**Table S3** Summary of best three models based on CWM and FD_Q_

| Response |  | Model 1 |  |  | Model 2 |  |  | Model 3 |  |  |
| --- | --- | --- | --- | --- | --- | --- | --- | --- | --- | --- |
|  |  | Selected traits | Estimates | rel. weight | Selected traits | Estimates | rel. weight | Selected traits | Estimates | rel. weight |
| Biomass |  | Intercept | -823.66 |  | Intercept | -1608.25 |  | Intercept | 355.85 |  |
|  | CWM | l.shoot | 127.78 |  | l.shoot | 129.34 |  | vert.leaf | -102.69 |  |
|  |  | vert.leaf | -140.21 |  | vert.leaf | -137.81 |  | SMF | 803.72 |  |
|  |  | N.leaf | 312.84 |  | N.leaf | 435.55 |  | biom:N | -5.66 |  |
|  |  | δ^15^N | -325.55 |  | δ^15^N | -372.78 |  | δ^15^N | -264.33 |  |
|  |  | life | 189.92 |  | root.type | 103.99 |  | life | 258.66 |  |
|  |  | l.rhythm | -96.54 |  | life | 229.62 |  | l.rhythm | -125.46 |  |
|  |  | #seed | -25.59 |  | l.rhythm | -110.50 |  |  |  |  |
|  | FD | δ^15^N | 40.77 | 1.00 | δ^15^N | 36.96 | 0.88 | δ^15^N | 41.46 | 1.00 |
|  |  |  |  |  | IMF | 48.09 | 1.00 |  |  |  |
|  |  |  | R^2^=0.818 |  |  | R^2^=0.826 |  |  | R^2^=0.813 |  |
| NE |  | Intercept | -1131.79 |  | Intercept | -1151.37 |  | Intercept | -1077.99 |  |
|  | CWM | vert.leaf | -163.17 |  | vert.leaf | -157.08 |  | vert.leaf | -152.22 |  |
|  |  | N.leaf | 308.69 |  | N.leaf | 324.31 |  | SLA | -3.69 |  |
|  |  | δ^15^N | -357.62 |  | δ^15^N | -330.53 |  | N.leaf | 315.65 |  |
|  |  | root.depth | 63.84 |  | root.depth | 40.74 |  | δ^15^N | -347.56 |  |
|  |  | root.type | 191.20 |  | root.type | 164.89 |  | root.depth | 59.32 |  |
|  |  | life | 150.13 |  | life | 182.77 |  | root.type | 187.47 |  |
|  |  | l.rhythm | -110.92 |  | l.rhythm | -106.39 |  | life | 147.90 |  |
|  |  |  |  |  |  |  |  | l.rhythm | -111.93 |  |
|  | FD | δ^15^N | 40.58 | 0.96 | δ^15^N | 39.20 | 0.87 | δ^15^N | 39.43 | 0.94 |
|  |  | clonal | 38.34 | 0.94 | clonal | 52.20 | 1.00 | clonal | 38.98 | 0.94 |
|  |  | IMF | 43.65 | 1.00 |  |  |  | IMF | 44.47 | 1.00 |
|  |  |  | R^2^=0.776 |  |  | R^2^=0.768 |  |  | R^2^=0.777 |  |
| CE |  | Intercept | 380.25 |  | Intercept | 912.18 |  | Intercept | 831.57 |  |
|  | CWM | clonal | -356.38 |  | root.type | -336.56 |  | δ^15^N | 385.94 |  |
|  |  |  |  |  |  |  |  | root.type | -387.97 |  |
|  |  |  |  |  |  |  |  | clonal | -220.06 |  |
|  |  |  |  |  |  |  |  | #seed | -56.00 |  |
|  | FD | N.leaf | 143.35 | 1.00 | N.leaf | 156.23 | 1.00 | N.leaf | 136.89 | 1.00 |
|  |  |  |  |  |  |  |  | life | 93.36 | 0.83 |
|  |  |  | R^2^=0.317 |  |  | R^2^=0.334 |  |  | R^2^=0.468 |  |
| SE |  | Intercept | -110.26 |  | Intercept | 26.71 |  | Intercept | 73.91 |  |
|  | CWM | δ^15^N | -484.97 |  | δ^15^N | -597.19 |  | δ^15^N | -653.33 |  |
|  |  | root.type | 349.31 |  | root.type | 391.91 |  | root.type | 385.91 |  |
|  |  | m.seed | 64.40 |  |  |  |  |  |  |  |
|  | FD | N.leaf | -106.48 | 0.98 | SMF | -66.32 | 0.78 | N.leaf | -117.38 | 1.00 |
|  |  | life | -111.11 | 1.00 | N.leaf | -106.69 | 0.99 | life | -115.79 | 0.99 |
|  |  |  |  |  | life | -108.23 | 1.00 |  |  |  |
|  |  |  | R^2^=0.396 |  |  | R^2^=0.397 |  |  | R^2^=0.383 |  |

For abbreviations of variable names see Table 2.
