# Supplementary material for: Using Plant Functional Traits to Explain Diversity–Productivity Relationships
Source: PLoS One. 2012 May 18;7(5):e36760. doi: 10.1371/journal.pone.0036760 (PMC3356333; doi:10.1371/journal.pone.0036760)
Supplement: Text S1 — Detailed method description on estimating the relative importance of functional traits. (PDF) [file pone.0036760.s004.pdf]

Appendix S1: Estimating the relative importance of functional traits

Calculation of all functional diversity indices is based on a functional trait matrix **T**,

$$\mathbf{T} = \begin{pmatrix} t_{11} & \cdots & t_{1k} \\ \vdots & \ddots & \vdots \\ t_{s1} & \cdots & t_{sk} \end{pmatrix}$$

describing each species as an element of a multidimensional trait space. Here t_{il} is the value of the trait l for species i, $(i = 1, \ldots, S; l = 1, \ldots, k)$.

In particular, Rao's quadratic diversity FD_Q is defined as

$$FD_Q = \sum_{i=1}^{S} \sum_{j=1}^{S} p_i p_j d_{ij} \ ,$$

where p_i and p_j are the relative abundances of species i and j, and d_{ij} describes the functional dissimilarity between species i and j, i.e., the distance between species i and j in the multidimensional trait space. Since any distance measure can be chosen, FD_Q in fact offers a huge variety of different diversity measures. We restrict our attention to one particular distance measure, the squared Euclidean distance, because this choice offers technical as well as interpretational advantages. Choosing

$$d_{ij} = \sum_{l=1}^{k} (t_{il} - t_{jl})^2$$

makes FD_Q additive with respect to traits:

$$\begin{aligned} FD_Q &= \sum_{i=1}^{S} \sum_{j=1}^{S} p_i p_j d_{ij} \\ &= \sum_{i=1}^{S} \sum_{j=1}^{S} p_i p_j \left(\sum_{l=1}^{k} (t_{il} - t_{jl})^2 \right) \\ &= \sum_{l=1}^{k} \left(\sum_{i=1}^{S} \sum_{j=1}^{S} p_i p_j (t_{il} - t_{jl})^2 \right) \ . \end{aligned}$$

Thus the contribution of different functional traits to the functional diversity of a community can easily be partitioned. Additionally it can be shown that

$$\sum_{i=1}^{S} \sum_{j=1}^{S} p_i p_j (t_{il} - t_{jl})^2 = 2 \sum_{i=1}^{S} p_i (t_{il} - \sum_{j=1}^{S} p_j t_{jl})^2 = 2 \ Var(t_l)$$

thus relating FD_Q to the sum of variances of individual traits, a commonly used measure of variability.

Another source of flexibility is the relative importance of particular traits in the calculation of FD_Q. Usually all traits are standardized before calculating dissimilarities, thus implicitely treating all functional traits as equally important. However, the assessment of the importance of different particular traits is often at the core of the scientific endeavour. The additive partitioning of FD_Q when based on the squared Euclidean distance offers a unique opportunity to estimate the relative importance of different traits.

To this end we consider a weighted trait matrix

$$T^\alpha = \begin{pmatrix} \alpha_1 t_{11} & \cdots & \alpha_k t_{1k} \\ \vdots & \ddots & \vdots \\ \alpha_1 t_{s1} & \cdots & \alpha_k t_{sk} \end{pmatrix}$$

with a priori unknown trait weights $\alpha_1, \ldots, \alpha_k$. The resulting FD_Q measure is a weighted sum of variances of individual traits:

$$\begin{aligned} FD_Q^\alpha &= \sum_{i=1}^{S}\sum_{j=1}^{S} p_i p_j \left(\sum_{l=1}^{k} (\alpha_l t_{il} - \alpha_l t_{jl})^2\right) \\ &= \sum_{l=1}^{k} \alpha_l^2 \left(\sum_{i=1}^{S}\sum_{j=1}^{S} p_i p_j (t_{il} - t_{jl})^2\right) \\ &= 2\sum_{l=1}^{k} \alpha_l^2 \, Var(t_l) \end{aligned}$$

A linear regression model, relating any community measure y_m (e.g. community biomass) to the functional diversity of the communities, $FD_{Q,m}$, can be written as:

$$\begin{aligned} y_m &= \beta_0 + \beta_1 FD_{Q,m} \\ &= \beta_0 + \beta_1 \sum_{l=1}^{k} \alpha_l^2 \left(\sum_{i=1}^{S}\sum_{j=1}^{S} p_i p_j (t_{il} - t_{jl})^2\right) \\ &= \beta_0 + \sum_{l=1}^{k} \left(\beta_1 \alpha_l^2\right) \left(\sum_{i=1}^{S}\sum_{j=1}^{S} p_i p_j (t_{il} - t_{jl})^2\right) \\ &= \beta_0 + \sum_{l=1}^{k} \gamma_l z_{l,m} \end{aligned}$$

The coefficients γ_l, and consequently the relative trait weights α_l, can therefore be estimated in a multiple linear regression model with predictors $z_{l,m}$ which describe the diversity of community m with respect to trait l. The particular form of the regression

coefficients $\gamma_l = \beta_1 \alpha_l^2$ implies an additional constraint - all γ_l must have the same sign. Estimation must therefore rely on least square estimation with box constraints (all γ_l positive or negative, respectively) available in standard statistical packages (we used the `quadprog` R package).

Because $k+1$ parameters are incorporated in the definition of the k regression coefficients γ_l, we need one further constraint to make the parameters identifiable. We fix the largest α_l to 1, thus defining

$$\begin{aligned}\beta_1 &= \max_{l=1,\ldots,k} \gamma_l \\ \alpha_l^2 &= \frac{\gamma_l}{\max_{l=1,\ldots,k} \gamma_l} = \frac{\gamma_l}{\beta_1} \ ,\end{aligned}$$

emphasizing the interpretation of α_l as the relative importance of traits l.

Due to trade-offs between traits there is obviously a certain amount of correlation between functional diversity measures associated with different traits. Thus the estimated relative weights are not fully independent estimates of relative importance and can become very imprecise if multicollinearity is severe.

Additional predictors (CWM, species richness) can easily be incorporated into the multiple linear regression model.
